# Interim Evaluation of the Tier 1 Program (Secondary 1 Curriculum) of the Project P.A.T.H.S.: First Year of the Full Implementation Phase

**DOI:** 10.1100/tsw.2008.5

**Published:** 2008-01-14

**Authors:** Daniel T. L. Shek, Hing Keung Ma, Rachel C. F. Sun

**Affiliations:** ^1^ Quality of Life Centre, Hong Kong Institute of Asia-Pacific Studies, The Chinese University of Hong Kong, Hong Kong; ^2^ Kiang Wu Nursing College of Macau, Macau; ^3^ Social Welfare Practice and Research Centre, The Chinese University of Hong Kong, Hong Kong; ^4^ Education Studies Department, Hong Kong Baptist University, Hong Kong

**Keywords:** interim evaluation, process evaluation, positive youth development program, Chinese adolescents

## Abstract

To understand the implementation quality of the Tier 1 Program (Secondary 1 Curriculum) of the Project P.A.T.H.S. (Positive Adolescent Training through Holistic Social Programmes) in the full implementation phase, 100 schools were randomly selected to participate in personal and/or telephone interviews regarding the quality of the implementation process of the Tier 1 Program. In the interviews, the participants described the responses of the students to the program, the perceived benefits of the program, the perceived good aspects of the program, and the areas requiring improvement, difficulties encountered in the implementation process, and perceived attributes of the worker-support scheme (“Co-Walker Scheme”). Results showed that most workers perceived that the students had positive responses to the program and the program was beneficial to the students. They also identified several good aspects in the program, although negative comments on the program design and difficulties in the implementation process were also recorded. Roughly half of the respondents had positive comments on the “Co-Walker Scheme”. In sum, the respondents generally regarded the program as beneficial to the students and they were satisfied with the Tier 1 Program (Secondary 1 Curriculum) in the full implementation phase, although some implementation difficulties were also expressed.

### INTRODUCTION

To promote holistic adolescent development, The Hong Kong Jockey Club Charities Trust earmarked HK$400 million for a positive youth development program entitled "P.A.T.H.S. to Adulthood: A Jockey Club Youth Enhancement Scheme" for junior secondary school students (Secondary 1 to 3) in Hong

Kong. "P.A.T.H.S." denotes Positive Adolescent Training through Holistic Social Programmes[1,2]. There are two implementation phases in this project – the experimental implementation phase and the full implementation phase. For the experimental implementation phase (January 2006 to August 2008), 52 secondary schools were invited to participate in the project with the objectives of accumulating experience in program implementation and familiarizing front-line workers with the program design and philosophy. In the 2006/07 school year, the programs were implemented on a full scale at the Secondary 1 level. In the 2007/08 school year, the programs were implemented at the Secondary 1 and 2 levels. In the 2008/09 school year, the programs will be implemented at the Secondary 1, 2, and 3 levels. The details of P.A.T.H.S. can be seen elsewhere[3].

In the Tier 1 Program, students in Secondary 1 to 3 participate in the program, normally with 20 h of training in the school year at each grade. The research team has developed a set of curriculum manuals, which include curriculum materials based on the 15 positive youth development constructs identified from the existing, successful, positive youth development programs: bonding, resilience, social competence, emotional competence, cognitive competence, behavioral competence, moral competence, self-determination, spirituality, self-efficacy, clear and positive identity, beliefs in the future, recognition for positive behavior, prosocial involvement, and prosocial norms[4]. In each grade, 40 teaching units each of 30-min duration were designed, based on the theoretical framework of positive youth development constructs, relevant research findings, and existing programs in both local and foreign contexts.

As adolescent development is influenced by the interactions between young people and their surrounding environment[5], the ecological perspective was adopted in P.A.T.H.S. by designing the teaching units that are intended to cultivate students' development in five different domains – individual, family, peer, school, and society. For example, some units in the Secondary 1 Curriculum were designed to promote students' relationships with their teachers and classmates, which help the Secondary 1 students adapt to their new school life. Moreover, as there are worrying trends and phenomena related to the development of adolescents in Hong Kong, such as mental health problems, abuse of psychotropic substances, adolescent suicide, school violence, and drop in family solidarity[6], some teaching units were designed to tackle some current youth issues, like (1) mental health problems, such as depressed moods and Internet addiction; (2) substance abuse and smoking; (3) heterosexual relationships; and (4) materialism. Furthermore, various kinds of teaching materials (e.g., student worksheets, PowerPoint, and soundtracks) were developed to facilitate the transmission of learning targets of the teaching units.

With reference to program planning and implementation, several mechanisms were used to evaluate the project. For the first year of the experimental implementation phase (2005/06), satisfactory evaluation findings were generated from different sources and different methods, including objective outcome evaluation[7], subjective outcome evaluation[8,9,10,11], qualitative evaluation[12], and process evaluation in term of observations[13]. As process evaluation is an indispensable part of program evaluation[14,15], an interim evaluation was also conducted during the program implementation process to gain more understanding of the reactions of the participants and workers to the program. Results showed that the workers had positive comments on the program, although the workers also encountered problems and difficulties in the implementation[16].

Meyer et al.[17] suggested that the comments of the participants and workers regarding the program implementation can act as a feedback loop for program refinement. Actually, the research team took the evaluation findings in the early stage of the experimental implementation phase into consideration, and made some modifications in the Secondary 1 Curriculum for the full implementation phase. For instance, some activities were slightly modified to cater to students' needs, some instructions were clearly presented to facilitate instructors' teaching, the number of student worksheets was reduced, artwork was produced and included in the worksheets, PowerPoint was included to attract students' attention, and a flash game was produced to enhance interaction and stimulation. In addition, the research team launched a "Co-Walker Scheme" in December 2006, which aimed to provide ongoing support and guidance to the participating schools. In the scheme, six co-walkers, who were the colleagues of the project, took care of the participating schools in the format of providing consultation and support, school visits, and observations. All these efforts were made with the purpose to enhance the quality of the curriculum manuals, the feasibility of the program in schools, and the morale of the front-line workers.

As some modifications were made in the Secondary 1 Curriculum and the implementation time of the first year of the full implementation phase (September 2006 to August 2007) was longer than that of the experimental implementation phase (January 2006 to August 2006), it was necessary to carry out an interim evaluation of the full implementation phase. Therefore, an interim evaluation was carried out to examine the views of the workers regarding the implementation of the Tier 1 Program of the Secondary 1 Curriculum, based on a random sample of schools who joined the first year of the full implementation phase in the 2006/07 school year.

### METHOD

#### Participants

In the full implementation phase in 2006/07, 207 schools joined the Secondary 1 Curriculum of P.A.T.H.S. Among these schools, 112 adopted the 20-h full program that involves 40 teaching units and 95 adopted the 10-h core program that involves 20 teaching units. Among these participating schools, 70 schools that joined the full program and 30 schools that joined the core program were randomly selected to join this study. In these selected schools, the instructors of the program (either school teachers or social workers) were invited to participate in face-to-face interviews on a voluntary basis during a school visit. If the respondents were not available for interviews during the school visit, they were invited to participate in telephone interviews or to fill in self-administered questionnaires and return via e-mail or fax. Among these 100 schools, 77 took part in face-to-face interviews, 15 participated in telephone interviews, and eight completed self-administered questionnaires. The respondents included 66 teachers and 45 social workers. The number of schools that participated in this research can be regarded as respectable, as about half of the participating schools of the project joined the interviews. Moreover, because the schools were randomly selected, the generalizability of the findings could be enhanced. These justifications satisfy Principle 2 in the implementation of qualitative evaluation research proposed by Shek et al.[18].

#### Procedures

According to Shek et al.[18], the procedures of a qualitative research should be clearly presented (Principle 3). As such, the procedures for data collection are systematically described below. The data collection was conducted between January and April 2007. As the full implementation phase took place from September 2006 to August 2007, January to April 2007 can be regarded as the midway point of the implementation process. While the presence of interviewers may affect the responses of the respondents (e.g., social desirability effect) in face-to-face interviews, it is unlikely to happen because the interviewers were the co-walkers of the interviewed schools with which a friendly rapport and mutual trust had been built. Also, the major advantage of face-to-face interviews is having the opportunity to clarify any doubts instantly. On the other hand, while telephone interviews and self-administered questionnaires have the problems of psychological distance and inability to observe the nonverbal cues of the respondents, their major advantage is efficiency in collecting the data within the time limit. In addition, follow-up calls could be arranged if there was a need to clarify the responses of the respondents. Therefore, these data collection methods have complemented each other.

A self-constructed, semi-structured interview guide with five closed-ended questions and seven open-ended questions was used to collect information on the program implementation process. The open-ended questions were:

- Question 1: What are the responses of the students to this program?
- Question 2: Do you think this program is beneficial to the students? If yes, what are the benefits?
- Question 3: What are the good aspects of the program?
- Question 4: Which areas of the program require improvement?
- Question 5: Have you encountered any difficulties during the program implementation process? If yes, what problems have you encountered?
- Question 6: What are your perceptions of the "Co-Walker Scheme"?
- Question 7: Do you have other opinions?

Informed consent was obtained from the respondents and they participated in the study in a voluntary manner. The interviews were conducted by six co-walkers, who were a colleague having a doctoral degree and five registered social workers with substantial working experience. After each interview, the interviewers were required to fill in the questionnaires. The completed questionnaires were then transcribed and analyzed.

### Data Analyses

For the quantitative data (close-ended questions), frequencies and percentages of responses were calculated. The qualitative data (open-ended questions) were analyzed using general qualitative analyses techniques[19]. There were three steps in the process. First, relevant raw codes were developed for words, phrases, and/or sentences that formed meaningful units at the raw responses level. Second, the codes were further combined to reflect higher-order attributes at the category of codes level. Third, the categories of codes were further analyzed to reveal the broader themes at the thematic level. For example, the response to "involved in games" at the raw response level could be subsumed under the category of "high student involvement", which could be further subsumed under the broad theme of "positive responses" of the students to the program (see Table 2). Following the principles of qualitative analyses[18], the raw data and categorized data were kept by a systematic filing system in order to ensure that the findings are auditable.

The qualitative data were coded and categorized by two trained research assistants, of which one has a Bachelor degree of Psychology and another has a Master degree of Social Work, which were further checked by the third author. In the present analyses, because the researchers designed the P.A.T.H.S. program, they were conscious of their own biases and expectations of the program to be effective. In addition, in order to minimize the possible biases involved, both intra- and inter-rater reliability on the coding was calculated. For intrarater reliability, a research assistant and the third author individually coded 20 randomly selected responses for each question. For inter-rater reliability, a research assistant with a Master degree and another research assistant who is a registered social worker, who were involved in the data collection and analyses, coded 20 randomly selected responses for each question without knowing the original codes given at the end of the scoring process with reference to the codes finalized by the first author. This met the Principles 4, 5, 6, 7, 8, and 9 of conducting a qualitative study advocated by Shek et al.[18].

## RESULTS

### Implementation Mode

Among the 100 interviewed schools, 50 adopted teaching mode 3 (i.e., 1-h per session) to implement the program, 18 adopted teaching mode 1 (i.e., a combination of 2.5-h per session and 1-h per session), 17 adopted teaching mode 2 (i.e., a combination of 30-min per session and 1-h per session), 13 adopted teaching mode 4 (i.e., 30-min per session), and two adopted another teaching mode designed by themselves. In the implementation, 46 schools incorporated the program into formal curriculum, such as Life Education, Liberal Studies, Moral and Civic Education, Religious Studies, and Integrated Humanities; 13 schools incorporated the program into class teachers' period; and eight schools incorporated the program into other time slots, such as morning assembly, weekly assembly, after school period, Saturday mornings, extracurricular activities, and day camps. For the remaining 33 schools, the program was incorporated into a combination of two or three of the following: formal curriculum, class teachers' period, and other time slots.

### Responses of the Students to the Program

As shown in Table 1, about 94 and 93% of the respondents reported that the students were involved and liked the program, respectively. A total of 230 meaningful units in three categories (i.e., positive responses, neutral responses, and negative responses) were formed to indicate the workers' perceptions of the students' responses to the Tier 1 Program (Table 2). Most of the informants perceived that students had positive responses (63.9%), such as "high student involvement" and "welcomed by students"; only two-fifths of them perceived negative responses (27.0%), such as "low student involvement" and "feeling bored", or neutral responses (9.1%), such as "students' responses were average". The intrarater agreement percentages were 100 and 95%, and the inter-rater agreement percentages were 95 and 90%, respectively.

### Perceived Benefits of the Program to the Students

As shown in Table 1, 94% of the respondents regarded the Secondary 1 Program to be slightly helpful, helpful, and very helpful to the students. Among 161 meaningful units of the perceived benefits of the program (Table 3), many of them pointed out that the program could "strengthen students' behavioral, cognitive, emotional and moral competence" (32.9%) and "build up interpersonal relationships" (21.7%), which were followed by "enhancing students' self-development" (6.8%), "facilitating students' learning" (6.2%), and "facilitating students' holistic development" (5.0%). There were 27.3% other responses, including "generally beneficial to students", "no to minimal benefits", "undecided", and "no comment". The intrarater agreement percentages were 95 and 90%, and the inter-rater agreement percentages were 100 and 95%, respectively.

### Positive Aspects of the Program and Areas that Require Improvement

There were 208 meaningful units regarding the good aspects of the program (Table 4). Except for three responses (1.4%) that were coded "none/no comment", the majority indicated having good "curriculum content and activity design" (42.8%) and good "teaching manuals and resources" (37.5%). For the remaining responses, there were 7.2% responses indicating that the "philosophy" underlying the project was good and 11.1% "other responses", e.g., "beneficial to teachers and students, and their relationships". The intrarater agreement percentages were 100 and 80%, and the inter-rater agreement percentages were 85 and 85%, respectively. On the other hand, there were 188 meaningful units concerning the aspects of the program required for improvement (Table 5). Except for six responses (3.2%) that indicated "none", about half of the responses indicated that the "curriculum content and activity design" needed to be modified (50.5%), and the remaining responses suggested improving the "teaching manuals and resources" (26.1%), and adjusting the "implementation time" (14.9%). There were 5.3% "other responses", such as suggesting "increase manpower/improve skills". The intrarater agreement percentages were 95 and 95%, and the inter-rater agreement percentages were 80 and 95%, respectively.

**TABLE 1**
**Instructors' Ratings of the Secondary 1 Curriculum (N and Percentage)**

1. Perceived degree of student involvement

<table>
<tr><th></th><th>Totally Not Involved</th><th>Not Involved</th><th>Involved</th><th>Totally Involved</th><th>No Response</th><th>Total</th></tr>
<tr><td>N</td><td>0</td><td>6</td><td>84</td><td>10</td><td>0</td><td>100</td></tr>
<tr><td>%</td><td colspan="2">6</td><td colspan="2">94</td><td>0</td><td>100</td></tr>
</table>

2. Perceived degree of students' liking of the Secondary 1 Curriculum

<table>
<tr><th></th><th>Strongly Dislike</th><th>Dislike</th><th>Like</th><th>Strongly Like</th><th>No Response</th><th>Total</th></tr>
<tr><td>N</td><td>0</td><td>5</td><td>89</td><td>4</td><td>2</td><td>100</td></tr>
<tr><td>%</td><td colspan="2">5</td><td colspan="2">93</td><td>2</td><td>100</td></tr>
</table>

3. Perceived degree of helpfulness of the Secondary 1 Curriculum

<table>
<tr><th></th><th>Unhelpful</th><th>Not Very Helpful</th><th>Slightly Helpful</th><th>Helpful</th><th>Very Helpful</th><th>No Response</th><th>Total</th></tr>
<tr><td>N</td><td>0</td><td>4</td><td>46</td><td>45</td><td>3</td><td>2</td><td>100</td></tr>
<tr><td>%</td><td colspan="2">4</td><td colspan="3">94</td><td>2</td><td>100</td></tr>
</table>

4. Perceived degree of workers' liking of the Secondary 1 Curriculum

<table>
<tr><th></th><th>Strongly Dislike</th><th>Dislike</th><th>Like</th><th>Strongly Like</th><th>No Response</th><th>Total</th></tr>
<tr><td>N</td><td>0</td><td>3</td><td>78</td><td>5</td><td>14</td><td>100</td></tr>
<tr><td>%</td><td colspan="2">3</td><td colspan="2">83</td><td>14</td><td>100</td></tr>
</table>

5. Perceived degree of workers' overall satisfaction of the Secondary 1 Curriculum

<table>
<tr><th></th><th>Very Dissatisfied</th><th>Dissatisfied</th><th>Slightly Dissatisfied</th><th>Slightly Satisfied</th><th>Satisfied</th><th>Very Satisfied</th><th>No Response</th><th>Total</th></tr>
<tr><td>N</td><td>0</td><td>1</td><td>7</td><td>23</td><td>62</td><td>3</td><td>4</td><td>100</td></tr>
<tr><td>%</td><td colspan="3">8</td><td colspan="3">88</td><td>4</td><td>100</td></tr>
</table>

### Difficulties Encountered During Program Implementation

As shown in Table 6, among 150 meaningful units of the difficulties encountered by the workers during program implementation, about half of the responses were related to difficulties in "administration and coordination" (32.7%) and "classroom and time management" (21.3%). About one-third of the responses were related to the difficulties in handling the "curriculum content and activity design" (13.3%), "students' responses" (11.3%), and "workload and stress" (10.0%). There were 10 responses (6.7%) that indicated "none/no comment" and 4.7% "other responses", such as having "not enough support from teachers/school". The intrarater agreement percentages were 100 and 100%, and the inter-rater agreement percentages were 80 and 90%, respectively.

**TABLE 2**
**Workers' Perceptions of the Responses of the Students to the Secondary 1 Curriculum**

| | Total Count (%) |
|---|---|
| Positive Responses | 147 (63.9) |
| ▪ High student involvement (e.g., "involved in games/role play", "enthusiastic", "eager to participate", "willing to answer questions", "active to voice out opinions") (N = 66) | |
| ▪ Welcomed by students (e.g., "liked the program", "looking forward to attending the lessons") (N = 29) | |
| ▪ Facilitated student learning (e.g., "students could learn something outside formal curriculum", "students were able to master the skills learned") (N = 15) | |
| ▪ High student interest (e.g., "interested in the topics", "the program/teaching materials could attract students) (N = 12) | |
| ▪ Other responses (e.g., "good responses", "open", "relaxed", "conscientious") (N = 25) | |
| Neutral Responses | 21 (9.1) |
| ▪ "It depends on the nature of students/topics/teachers" (N = 10) | |
| ▪ "Students' responses were average" (N = 11) | |
| Negative Responses | 62 (27.0) |
| ▪ Low student involvement (e.g., "not involved", "passive", "not willing to participate") (N = 10) | |
| ▪ Feeling bored (e.g., "students were bored by some topics/audio-visual materials/repeated content", "students considered the content was simple/childish/did not match their needs") (N = 24) | |
| ▪ Incapability (e.g., "students could not understand some topics", "students could not carry out group discussion") (N = 12) | |
| ▪ Other responses (e.g., "poor discipline", "diminishing student motivation", "resistant", "students did not like to discuss/do worksheets") (N = 16) | |
| Total | 230 (100) |

### Perceptions of the "Co-Walker Scheme"

A total of 114 meaningful units in five categories (i.e., positive responses, neutral responses, negative responses, none or no comment, and other responses) were formed to indicate the workers' perceptions of the "Co-Walker Scheme" (Table 7). Except for 39 responses (34.2%) that indicated "none/no comment", half of the total responses were positive (50.0%), such as "it can strengthen communication", which was followed by other responses (10.0%), such as "it is hoped that the research team can receive school comments and revise the program". There were only 3.5% negative responses, such as "it has no practical use", and 1.8% neutral responses, such as "it's OK". The intrarater agreement percentages were 100 and 95%, and the inter-rater agreement percentages were 85 and 80%, respectively.

### Other Opinions of the Project

As shown in Table 1, there were about 83 and 88% of the respondents showing they liked the program and felt satisfied, respectively. At the same time, there were 81 meaningful units regarding the respondents' opinions of the project (Table 8), of which 35.8% indicated "none", 22.2% comments related to "curriculum content and activity design", 17.3% comments related to "school administration and coordination", and 24.7% were "other comments and suggestions" on training, students responses, and resources. The intrarater agreement percentages were 95 and 85%, and the inter-rater agreement percentages were 95 and 80%, respectively.

**TABLE 3**
**Workers' Perceptions of the Benefits of the Secondary 1 Curriculum**

| | Total Count (%) |
|---|---|
| Facilitating Students' Holistic Development<br>▪ "The content is broad/related to daily life" (N = 4)<br>▪ "It helps students' growth/holistic development" (N = 4) | 8 (5.0) |
| Building Up Interpersonal Relationships<br>▪ "Students learn how to choose/get along with friends" (N = 15)<br>▪ "It enables students to communicate with others" (N = 16)<br>▪ "It facilitates teacher-student/parent-child relationships" (N = 4) | 35 (21.7) |
| Strengthening Students' Behavioral, Cognitive, Emotional, and Moral Competence<br>▪ "It promotes students' thinking/reflection skills" (N = 15)<br>▪ "It promotes students' emotional management" (N = 11)<br>▪ "It promotes students' moral development" (N = 18)<br>▪ "It promotes students' refusal skills/presentation skills" (N = 9) | 53 (32.9) |
| Enhancing Students' Self-Development<br>▪ "It builds up students' self-confidence/efficacy/character" (N = 7)<br>▪ "It promotes students' self-development" (N = 4) | 11 (6.8) |
| Facilitating Students' Learning<br>▪ "It facilitates students' learning attitudes/knowledge and applications" (N = 7)<br>▪ "Students have become active in self-discourse and discussion" (N = 3) | 10 (6.2) |
| Other Responses<br>▪ Generally beneficial to students (N = 12)<br>▪ No to minimal benefits (N = 17)<br>▪ Undecided (N = 13)<br>▪ No comment (N = 2) | 44 (27.3) |
| Total | 161 (100) |

### DISCUSSION

The primary purpose of this paper is to report interim evaluation findings on the implementation of the Tier 1 Program (Secondary 1 Curriculum) of the Project P.A.T.H.S. in the first year of the full implementation phase (2006/07 academic year). Several phenomena could be highlighted from the present findings. First, the respondents felt that most of the students had positive responses to the program, with over 90% of them reporting that the students were involved and liked the program. Second, over 90% of the respondents rated the program as helpful to students, with a majority of them perceiving the program as beneficial to the students. Third, many respondents liked the program (83%) and were satisfied with the program (88%). These findings, based on the first year of the full implementation phase, basically reinforced the findings arising from the objective outcome evaluation[7], subjective outcome evaluation[8,9,10,11], qualitative evaluation[12], and process evaluation[13] in the experimental implementation phase that the program was helpful to the students, and both the students and workers had positive perceptions of the program.

As the implementation time of the full implementation phase was longer, no respondents reported having the problem of too short program implementation time as in the experimental implementation phase. However, although the respondents appreciated the program, they also identified areas requiring improvement and difficulties encountered in the implementation process. This observation is puzzling, particularly in view of the fact that some modifications had been made in the Secondary 1 Curriculum of

**TABLE 4**
**Workers' Perceptions of the Positive Aspects of the Secondary 1 Curriculum**

| | **Total Count (%)** |
|---|---|
| None/No Comment | 3 (1.4) |
| Teaching Manuals and Resources | 78 (37.5) |
| ▪ Diverse and good teaching aids (e.g., "it has multimedia resources", "the design of the teaching aids are attractive") (N = 21) | |
| ▪ Clear teaching plans (e.g., "teaching plans are clearly presented", "teaching plans are resourceful") (N = 36) | |
| ▪ Other responses (e.g., "sufficient resources", "flexible", "user-friendly") (N = 21) | |
| Curriculum Content and Activity Design | 89 (42.8) |
| ▪ Good curriculum content (e.g., "diverse topics", "rich content", "comprehensive coverage", "structural", "flexible", "it meets students' needs") (N = 48) | |
| ▪ Good activity design (e.g., "there are different activities", "the activity design is active/creative/rich/interesting / stimulating") (N = 41) | |
| Philosophy | 15 (7.2) |
| ▪ Good positive youth development constructs (e.g., "bonding", "recognition for positive behavior", "moral competence") (N = 6) | |
| ▪ Good theoretical basis (e.g., "good theoretical framework") (N = 9) | |
| Other Responses | 23 (11.1) |
| ▪ Beneficial to teachers and students, and their relationships (N = 13) | |
| ▪ Students welcomed the project/had good discipline (N = 3) | |
| ▪ Having good instructors/school support (N = 3) | |
| ▪ Having support from the project/appreciation to the project (N = 4) | |
| Total (%) | 208 (100) |

the full implementation phase. At any rate, the perceived difficulties and problems expressed by the respondents are regarded as invaluable pointers on how the Tier 1 Program (e.g., curriculum content and activity design), school administration, and coordination could be further improved. Consistent with the spirit of reflective practice, the comments collected and recorded would be seriously considered by the research team, although it is noteworthy that there could be different interpretations of the findings.

Regarding the difficulties and problems encountered during the process, there are several factors that contribute to these observations. First, because only a small proportion of the interviewed schools had participated in the experimental implementation phase (13%), it was the first time for a majority of the interviewed schools to implement a new positive youth development program in their existing curriculum, and thus the difficulties in administration and coordination, such as time-table arrangement and manpower deployment, were expected. Second, utilizing the curricula approach to implement a positive youth development program is a relatively new experience to workers in Hong Kong. As such, teachers and social workers need time to prepare the lessons and adjust to the mode of teaching (e.g., using games and interactive teaching methods), which is different from the traditional didactic form of teaching. In fact, under the influence of traditional Chinese beliefs, which emphasize the superior role of the teachers[20], some teachers might have difficulty "playing" with the students and sharing their experiences.

Third, because it is not common for social workers and teachers to use teaching manuals to deliver the program, they might find the program inflexible. In addition, because the program was conducted by social workers in collaboration with the teachers, they might need time to make the collaboration more streamlined. Fourth, overenthusiasm of the workers may account for the perceived time pressure experienced by the workers. It is noteworthy that activities of the teaching units had been field-tested where

**TABLE 5**
**Workers' Perceptions of the Aspects of the Secondary 1 Curriculum that Require Improvement**

| | **Total Count (%)** |
|---|---|
| None | 6 (3.2) |
| Curriculum Content and Activity Design | 95 (50.5) |
| ▪ "The content is too board/too abstract/too difficult/too simple/does not match students' interests or competence" (N = 51) | |
| ▪ "Too many activities/add more activities/increase variety" (N = 12) | |
| ▪ "Some topics/activities/growth puzzles are repetitive" (N = 16) | |
| ▪ Other responses (e.g., "the activity format is difficult to carry out", "teaching sequences can be better arranged", "increase flexibility") (N = 16) | |
| Teaching Manuals and Resources | 49 (26.1) |
| ▪ "Add more multimedia teaching aids/information" (N = 13) | |
| ▪ "Worksheets are too many/too difficult/too simple" (N = 13) | |
| ▪ "PowerPoint/teaching materials are boring/simple/difficult" (N = 8) | |
| ▪ "CD-ROM/teaching manuals are not useful/user-friendly/difficult to follow" (N = 5) | |
| ▪ Other responses (e.g., "to have an English version", "to have student handbooks", "to have WORD document version") (N = 10) | |
| Implementation Time | 28 (14.9) |
| ▪ "There is insufficient time for running the activities" (N = 11) | |
| ▪ "Time is pressing/often overruns" (N = 15) | |
| ▪ Other responses (e.g., "it is difficult to arrange a time-table") (N = 2) | |
| Other Responses | 10 (5.3) |
| ▪ "Increase manpower/improve skills" (N = 4) | |
| ▪ "Training is not helpful" (N = 2) | |
| ▪ "Workers need to tailor-make the program for students" (N = 1) | |
| ▪ "The degree of experiential learning depends on school environment" (N = 1) | |
| ▪ "Need longer-term resources support" (N = 1) | |
| ▪ "Need to incorporate into formal curriculum" (N = 1) | |
| Total | 188 (100) |

a worker should normally have sufficient time to deliver the designed activities. In addition, the teaching units had been recategorized into primary activities (i.e., activities that must be completed) and secondary activities (i.e., activities that should be completed if time is available). It might be because the workers were eager to run all the designed activities to meet the learning targets within the time limit, and thus they need to adjust their time management. Fifth, from the case study and informal contact with the workers, it was realized that probably because of administrative difficulties, some workers who had not received training were involved in the program implementation. This may also contribute to the observed situation. Sixth, because the perceived difficulties were strikingly similar to the findings of the experimental implementation phase[16], the related findings suggest the possibility of operation of response sets. As Chinese people subscribe to the "doctrine of the mean", it is not surprising to see that the program implementers described both positive features and problems of the program. Finally, P.A.T.H.S. was launched at a time where there was mounting frustration and dissatisfaction with the education reform after the handover in 1997[21,22]. In addition, with the introduction of the Lump Sum Grant system in the social welfare sector in Hong Kong, social workers have to compete for resources, which has adversely affected their morale. Against this political background, it is not surprising to find that some colleagues were basically negative to P.A.T.H.S.

**TABLE 6**
**Workers' Perceptions of the Difficulties Encountered in Secondary 1 Curriculum Implementation**

| | **Total Count (%)** |
|---|---|
| None/No Comment | 10 (6.7) |
| Administration and Coordination | 49 (32.7) |
| ▪ "It was difficult to arrange a time-table" (N = 13) | |
| ▪ "There were difficulties in arranging manpower/cooperation" (N = 17) | |
| ▪ "The physical classroom setting was limited for running activities" (N = 13) | |
| ▪ Other responses, (e. g., "not enough resources", "the school computer system did not function well) (N = 6) | |
| Workload and Stress | 15 (10.0) |
| ▪ "Teachers had to spend time for preparation/workload was heavy" (N = 12) | |
| ▪ "Teachers were stressful" (N = 3) | |
| Classroom and Time Management | 32 (21.3) |
| ▪ "It was difficult to manage a large class in running activities" (N = 10) | |
| ▪ "There was insufficient time to run the program" (N = 14) | |
| ▪ "Time was pressing" (N = 8) | |
| Curriculum Content and Activity Design | 20 (13.3) |
| ▪ "The content was too much/too abstract/not attractive" (N = 9) | |
| ▪ "Teaching aids were not attractive/too many worksheets/growth puzzles were repetitive and too large" (N = 11) | |
| Students' Responses | 17 (11.3) |
| ▪ "Classroom discipline was a problem" (N = 3) | |
| ▪ "Students were not attentive/not involved/not serious" (N = 8) | |
| ▪ "Students' levels of competence varied/their written ability was poor" (N = 6) | |
| Other Responses | 7 (4.7) |
| ▪ "Not enough support from teacher/school" (N = 2) | |
| ▪ "Teachers did not acquire adequate skills to run activities" (N = 1) | |
| ▪ "Students' performance was affected by teacher-students relationships/students' own emotion and behavior/examination) (N = 3) | |
| ▪ "Involved too much time" (N = 1) | |
| Total | 150 (100) |

Regarding the "Co-Walker Scheme", because it had just been launched for 1–4 months at the time of data collection, it is understandable that about one-third of the respondents had no comment or could not comment on it. Nonetheless, the positive responses (50%) indicated that the scheme was welcomed by workers and the obviously low negative comments are encouraging signs.

Following the Principle 10 of conducting a qualitative study[18], several possible alternative explanations were put forward for the present findings. First, the findings can be explained in terms of demand characteristics. However, this explanation is not likely because the respondents were encouraged to voice their views without restriction and negative voices were, in fact, heard. The second alternative explanation is that the findings are due to selection bias. However, this argument is not strong because the schools were randomly selected. The third alternative explanation is that the positive findings are due to ideological biases of the researchers. As several safeguards were used to reduce biases in the data collection and analysis process, this possibility is not high. Actually, multiple colleagues were involved, which provided checking for individual biases. In addition, intra- and inter-rater reliability checks were carried out to safeguard consistency in coding.

**TABLE 7**
**Workers' Perceptions of the "Co-Walker Scheme" (Secondary 1 Curriculum)**

| | Total Count (%) |
|---|---|
| None/No Comment | 39 (34.2) |
| Positive Responses | 57 (50.0) |
| ▪ "It is generally fine/good/appreciated" (N = 9) | |
| ▪ "It is supportive" (N = 9) | |
| ▪ "It can strengthen communication" (N = 20) | |
| ▪ "It enables understanding to the needs of front-line workers" (N = 8) | |
| ▪ "The co-walkers/workers can know more about the program implementation in different schools" (N = 10) | |
| ▪ "It enhances program improvement" (N = 1) | |
| Neutral Responses | 2 (1.8) |
| ▪ "It is OK" (N = 2) | |
| Negative Responses | 4 (3.5) |
| ▪ "It has no practical use" (N = 3) | |
| ▪ "Teachers felt pressured when co-walker conducted the visit" (N = 1) | |
| Other Responses | |
| ▪ "It is hoped that it can provide a platform for sharing" (N = 2) | 12 (10.5) |
| ▪ "It is hoped that the research team can receive school comments and revise the program" (N = 5) | |
| ▪ "It is hoped that the scheme can start earlier when term starts" (N = 3) | |
| ▪ "It is important not to increase workload" (N = 1) | |
| ▪ "It is hoped that co-walkers can provide training to teachers" (N = 1) | |
| Total | 114 (100) |

According to Principle 12 proposed by Shek et al.[18], several limitations of the study are described below. First, because each interview was brief (which was reasonable for a large-scale interim evaluation) and some data were collected through self-administrated questionnaires, the researchers were not able to collect in-depth information and construct "thick descriptions". Therefore, in-depth individual or focus group interviews would be helpful to further understand the subjective experiences of the program participants. Second, although other mechanisms of evaluation, such as objective outcome evaluation and subjective outcome evaluation have been carried out for the full implementation phase, the inclusion of case studies that aim to explore the factors facilitating or impeding the program implementation is needed. Despite these limitations, this study provides interim evaluation findings supporting the positive nature of P.A.T.H.S. and its effectiveness in promoting holistic youth development among Chinese adolescents in Hong Kong in the first year of the full implementation phase.

A survey of the literature on positive youth development shows that attempts to conduct process evaluation in the form of interim evaluation are not prevalent[15,23,24]. In particular, with the exception of P.A.T.H.S., interim evaluation findings are seldom documented in scientific publications in different Chinese contexts. As such, with gradual accumulation of the interim evaluation findings based on randomly selected schools in the project, the related findings definitely contribute to the Chinese literature on positive youth development programs. This is a very important step as far as evidence-based human services in different Chinese communities is concerned[25].

**TABLE 8**
**Other Opinions of the Project (Secondary 1 Curriculum)**

| | **Total Count (%)** |
|---|---|
| None | 29 (35.8) |
| Curriculum Content and Activity Design | 18 (22.2) |
| ▪ "The content is too abstract/too much/loose/repetitive" (N = 7) | |
| ▪ "It needs attractive teaching aids/simple games" (N = 6) | |
| ▪ "It needs more flexibility/alternatives" (N = 3) | |
| ▪ Other responses (e.g., "increase experiential learning element") (N = 2) | |
| School Administration and Coordination | 14 (17.3) |
| ▪ "It was difficult to arrange manpower" (N = 4) | |
| ▪ "There were difficulties in implementing Tier 2 Program" (N = 4) | |
| ▪ "It needs cooperation between school and agency" (N = 2) | |
| ▪ Other responses (e.g., "it needs time to adjust the curriculum", "the Co-Walker Scheme is appreciated") (N = 4) | |
| Other Comments and Suggestions | 20 (24.7) |
| ▪ Training (e.g., "training workshops are not enough/not useful", "workshops are effective", "it is suggested to arrange training workshops after term starts") (N = 5) | |
| ▪ Student responses (e.g., "they liked Tier 1/Tier 2 Program" "they did not like Tier 2 Program", "they were not serious") (N = 5) | |
| ▪ Resources (e.g., "limited resources", "helpful resources", "increase monitoring/instructions on using the funds") (N = 4) | |
| ▪ Other responses (e.g., "to create a platform for sharing", "teachers' support for the program is important", "to deepen the curriculum content") (N = 6) | |
| Total | 81 (100) |

### ACKNOWLEDGMENTS

The preparation for this paper and the Project P.A.T.H.S. were financially supported by the Hong Kong Jockey Club Charities Trust.
